# A Case of Intertwin Membrane Hemorrhage with Spontaneous Resolution

**DOI:** 10.1155/2019/3757454

**Published:** 2019-12-23

**Authors:** Lily Criscione, Kristen Elmezzi, Saioa Torrealday, Barton C. Staat, Kimberly Hickey

**Affiliations:** ^1^Walter Reed National Military Medical Center, 8901 Rockville Pike, Bethesda, MD 20889, USA; ^2^Uniformed Services University, 4301 Jones Bridge Rd., Bethesda, MD 20814, USA

## Abstract

Vaginal bleeding during pregnancy places women at increased risk of spontaneous abortion. Etiologies for threatened and spontaneous abortions have been well studied, but there is little information on intertwin membrane hemorrhage. We present a patient with a multiple gestation pregnancy who experienced first trimester vaginal bleeding with visualization and subsequent rapid resolution of an intertwin membrane hemorrhage. The patient had an otherwise normal pregnancy until the third trimester when she developed preeclampsia with severe features and elected for a primary cesarean section at 35 + 5 weeks. The implications of an intertwin membrane hemorrhage are not well understood, although there could be a possible correlation between the hemorrhage and the ultimate progression to preeclampsia with severe features. Despite the final diagnosis, the patient did not have any noticeable complications due to the hemorrhage both when it was discovered and in the weeks following its discovery.

## 1. Introduction

Vaginal bleeding during pregnancy places women at increased risk of spontaneous abortion. Etiologies for threatened and spontaneous abortions have been well studied, but there is little information on intertwin membrane hemorrhage. Intertwin membrane hemorrhage is an obstetric condition that is rarely observed; in fact, there is only one other case reported in the literature [1]. Equally to the pathophysiology of an abruption, one could postulate that an intertwin hemorrhage could increase the patient's risk of developing preterm labor or preterm prelabor rupture of membranes, as well as intrauterine growth restriction, oligohydramnios, or preeclampsia.

## 2. Case

A 33-year-old gravida 1 successfully conceived after ovulation induction with letrozole and intrauterine insemination. The patient's medical history was significant only for migraines. Her surgical history was notable for a laparoscopic right salpingo-oophorectomy and appendectomy for chronic right-sided pelvic pain.

**Figure 1:**
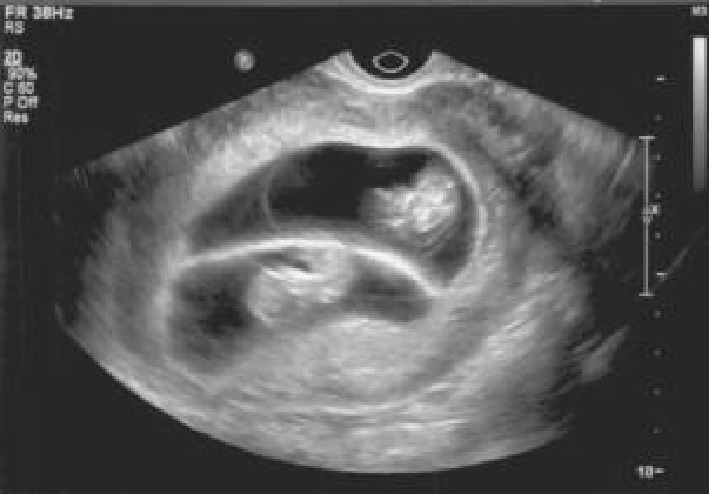
Patient at 10 + 1 weeks EGA with no evidence of abnormally thickened membrane.

The patient presented for an ultrasound at 5 + 2 weeks, which revealed 3 gestational sacs; however, only 2 contained yolk sacs. An ultrasound at 6 + 2 weeks confirmed a dichorionic twin pregnancy with 2 gestational sacs, yolk sacs, and fetal poles. At 9 + 2 weeks, she experienced vaginal bleeding followed by a second episode 4 days later. On both occasions, she was seen in the emergency room where ultrasound not only confirmed a viable twin pregnancy but also demonstrated a normal dividing membrane and no evidence of subchorionic bleeding. There was no intertwin hemorrhage noted. She continued to experience vaginal bleeding, yet subsequent evaluations were reassuring (Figure 1).

**Figure 2:**
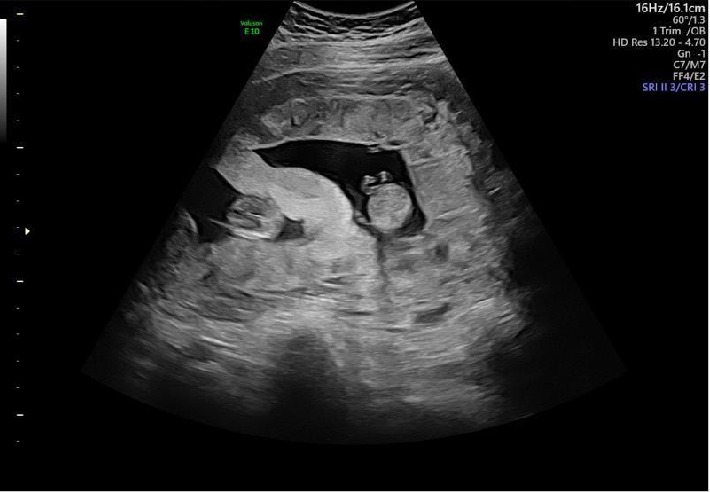
Patient at 11 + 0 weeks EGA with intertwin membrane hemorrhage, 15 mm in thickness.

The patient presented to perinatology for her nuchal translucency ultrasound at 11 weeks. By then, her vaginal bleeding had ceased; she denied contractions, cramping, and vaginal discharge. Her ultrasound showed a 15.0 mm intertwin membrane hemorrhage (Figure 2). The recommended baby aspirin was held, and the patient was given bleeding and preterm labor precautions.

**Figure 3:**
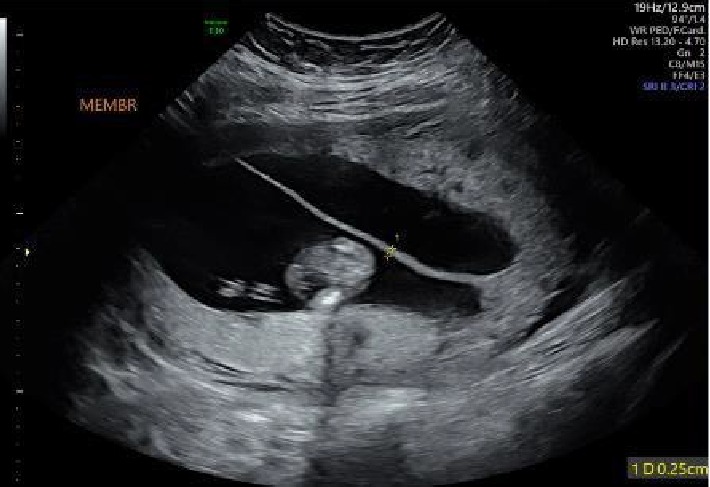
Patient at 13 + 3 weeks EGA with reabsorbed hemorrhage. Thickness now 2.5 mm.

When she returned at 13 + 3 weeks for follow-up, the hemorrhage had resolved; the spanning membranes measured 2.5 mm (Figure 3). She remained asymptomatic and denied vaginal bleeding. At her 16-week assessment, her imaging continued to be reassuring. The pregnancy progressed normally until 35 + 2 weeks, when she demonstrated mild hypertension. ALT and AST were 44 and 36, respectively; she was otherwise asymptomatic. The next day her AST and ALT were 58 and 78, and she was diagnosed with preeclampsia with severe features. Although she was offered an induction of labor, she underwent an elective primary cesarean section at 35 + 5 weeks following steroid administration for fetal lung maturity; estimated blood loss was 1 L. The cesarean was productive of two viable female infants with twin A of APGARS 8/8, weighing 2510 grams and twin B of APGARS 5/7/8, weighing 2300 grams. The twins had demonstrated appropriate growth throughout pregnancy. The patient completed a 24-hour course of magnesium following delivery; she received a transfusion of 2 units of packed red blood cells due to symptomatic anemia. She was discharged with both infants on postoperative day 5.

Upon inspection of the placentas at time of surgery, gross clot remnants between the twins' membranes were not appreciated. The pathology report showed diamniotic dichorionic nonfused placentas with an organized, dividing membrane hematoma. Placenta A's chorionic plate vessels demonstrated a possible eosinophilic or T-cell vasculitis. Neither decidual arteriopathy nor chorionic villitis was identified. Per the report, on the membrane between placenta A and B existed an area of confluent subchorionic fibrin deposition that measured 4.5 × 4.0 cm in diameter; no areas of acute hemorrhage or hematoma formation were identified.

## 3. Discussion

On review, there is only one other case report identifying a patient with an intertwin membrane hemorrhage visualized on ultrasound. Nevertheless, that patient's pregnancy was viable at 33 + 1 weeks when the hemorrhage was noted, and she was ultimately diagnosed with an abruption that was determined to be unstable, necessitating an emergency cesarean section. The patient did not develop subsequent sequelae from the bleed, such as preeclampsia [1]. That patient contrasts with our case, in which the patient was diagnosed with a stable hemorrhage that resolved with little discernable complications for the pregnancy. Yet with the eventual diagnosis of preeclampsia with severe features, one could postulate that the hemorrhage increased our patient's risk. Overall, both cases demonstrate ultrasound visualization of bleeding into the intertwin membrane space; however, the significance of the hemorrhages differ, in that the prior case demonstrated an abruption necessitating emergent delivery, whereas our patient, though a marginal abruption could be the underlying cause of the hemorrhage, had a bleed that was stable and ultimately resolved, enabling her to carry the pregnancy to the third trimester [1].

It is possible that when intermembrane hemorrhages occur they resolve quickly, leaving their incidence difficult to predict. Our patient was being monitored closely by multiple subspecialists and had frequent ultrasounds assessing her pregnancy's progression, increasing the opportunity of detecting transient abnormalities. With the increased use of infertility treatment modalities compounded by the increase incidence of multiple gestations within this population, perhaps more cases of intermembrane hemorrhage will be diagnosed and the sequelae will become clearer.

This irregularly thickened 15.0 mm dividing membrane was not a normal variant, as the vast majority of dichorionic twins show thickness between 1.9 and 3.6 mm [2, 3]. Differential diagnosis could include a circumvallate placenta, an amniotic band, uterine synechiae, uterine congenital abnormalities, or membranes from a vanishing twin. Due to the increased thickness, echogenicity, and relative homogeneity of the region in question as well as the transient nature, all the aforementioned possibilities are less likely than simply a hemorrhage collecting between the twins' amniotic borders.

The question that remained for our patient was the implications of the hemorrhage. She was counseled on premature preterm rupture of membranes and preterm contractions, as blood is a possible uterine irritant. Similar to the pathophysiology of an abruption, a hemorrhage in the intrauterine space can promote the release of thrombin, which acts as a uterotonic agent [4, 5]. Furthermore, thrombin ignites the inflammation cascade, which can cause further vascular degradation and possible rupture of membranes [6, 7]. Marginal abruption may not require an immediate delivery, but can result in other complications such as oligohydramnios and intrauterine growth restriction, neither of which our patient demonstrated [8]. Additionally, early vaginal bleeding in twin pregnancy has been shown to increase the overall gestation's risk of abruption, rupture of membranes, and preterm birth at less than 34 weeks [9]. Fortunately, the patient did not display preterm labor during her subsequent clinic visits, and the spontaneous resolution and reabsorption of the blood possibly returned her overall risk of preterm delivery to its baseline in a twin pregnancy [10]. Likewise, the twin pregnancy increased the patient's baseline risk for preeclampsia. The progression to preeclampsia with severe features could be correlated to the intertwin membrane hemorrhage as well, as a utero-placental malformation that allowed for the original bleed could predispose the patient to a subsequent placental disorder such as preeclampsia. Studies have shown that a history of abruption in a prior pregnancy can increase one's risk of developing preeclampsia in a following pregnancy [11].

In conclusion, intermembrane hemorrhage may be more commonplace than documented. There is a knowledge gap in regard to the prevalence of intermembrane hemorrhage and potential associated complications. Based on this case, we would recommend that intermembrane hemorrhages be managed conservatively with frequent follow-up ultrasounds and assessments for preterm labor, preterm prelabor rupture of membranes, oligohydramnios, intrauterine growth restriction, and preeclampsia. Regardless of the ambiguity that surrounds the findings and outcomes, this case is noteworthy, as it can offer guidance and counseling for future patients with intertwin membrane hemorrhages.

## Supplementary Materials

Supplementary Materials A patient with a twin gestation developed an intertwin membrane hemorrhage during the late first trimester that spontaneously resolved with little consequence for the pregnancy until the third trimester when she had preeclampsia with severe features and delivered via cesarean section. We review her case and images in the video.
